# Towards the elimination of infectious HPV: exploiting CRISPR/Cas innovations

**DOI:** 10.3389/fcimb.2025.1627668

**Published:** 2025-08-04

**Authors:** Wei Liu, Yingyan Jiang, Cheng Wang, Min Wang, Wei Zhang, Haiying Ren, Shiyan Xu, Jinjing Qin, Pan Liu, Lianhai Jin, Donghai Zhao

**Affiliations:** ^1^ College of Laboratory Medicine, Jilin Medical University, Jilin, China; ^2^ Department of Obstetrics and Gynecology, Affiliated hospital of Jilin medical university, Jilin, China; ^3^ College of Basic Medicine, Jilin Medical University, Jilin, China; ^4^ Department of Obstetrics and Gynecology, The Second Hospital of Jilin University, Changchun, China; ^5^ Low Pressure and Low Oxygen Environment and Health Intervention Innovation Center, Jilin Medical University, Jilin, China; ^6^ Psychiatric Research Unit III, Jilin Sixth People’s Hospital, Jilin, China

**Keywords:** HPV, CRISPR/Cas, gene editing, viral genome targeting therapy, HPV-associated diseases

## Abstract

HPV has been conclusively associated with various human malignancies, making the development of prevention and treatment strategies for HPV-induced diseases a high priority. Currently, primary prevention methods include HPV immunization and routine screening, which significantly reduce the risk of HPV transmission. However, for patients diagnosed with invasive, advanced, or recurrent malignancies, non-virus-specific therapies frequently lead to drug resistance and adverse effects, resulting in minimal improvement in treatment efficacy for numerous patients. Viral genome-targeting therapy is emerging as a promising avenue for the future management of HPV infections. With the rapid advancement of genetic modification technologies, the CRISPR/Cas system has demonstrated significant potential in treating viral infections. Its ability to selectively target and edit viral genomes for elimination positions it as a highly effective approach for combating HPV. This review will explore the functions and applications of the CRISPR/Cas system as an innovative therapy for HPV. We will illustrate the prospective efficacy of CRISPR/Cas as a groundbreaking and promising cure for HPV infections, while also addressing the opportunities and challenges associated with this novel approach.

## 1 Introduction

Globally, Human Papillomavirus (HPV) ranks as a leading sexually transmitted pathogen, exhibiting widespread prevalence through transmission via skin, genital, and oral contact. Over 200 genotypes have been identified, categorized as either high-risk or low-risk types based on their oncogenic potential (Wiley et al., 2002). High-risk HPV infections significantly contribute to the development of at least 40 different human malignancies, including cervical, anal, penile, and oropharyngeal cancers (Haedicke and Iftner, 2013; Ljubojevic and Skerlev, 2014). Notably, nearly 90% of squamous cell carcinomas of the anus are attributed to HPV, and warty carcinomas of the vulva and penis frequently test positive for the virus (Alemany et al., 2016, 2015; Faber et al., 2017). Cervical cancer, which is almost universally associated with HPV, poses a significant health threat. Data from the Bruni et al. (2023) indicate that an estimated 2.97 billion females aged 15 years and older are at risk of cervical cancer globally. Despite global vaccination initiatives against HPV, approximately 604,000 new cases of cervical cancer are reported each year, along with an annual death toll of around 342,000 from this disease. Consequently, the prevention and management of HPV remain critical challenges in global public health (Bruni et al., 2023). Effective treatment and control of HPV infections are essential for eradicating the virus, thereby preventing associated diseases, improving the quality of life for those affected, and alleviating healthcare burdens. Viral genome-targeting therapy emerges as a potential avenue for the future management of HPV infections. As genetic modification technology rapidly advances, the CRISPR/Cas system has shown considerable promise in treating viral infections. Its ability to selectively target and edit viral genomes for elimination positions it as a highly promising approach for combating HPV. This review will explore the functions and applications of the CRISPR/Cas system as an innovative therapy for HPV. We will demonstrate the prospective efficacy of CRISPR/Cas as a groundbreaking and promising treatment for HPV infections while addressing both the opportunities and challenges associated with this novel approach.

## 2 Etiology and carcinogenic mechanism of HPV

HPV encompasses a vast family of viruses, which are classified based on genetic variations into hierarchical levels: genus, species, type, variant lineage, and sublineage (Burk et al., 2013; Doorbar et al., 2012). Typically, HPV is categorized into five genera: α, β, γ, μ, and ν, distinguished by their nucleotide sequence homologies and the specific pathogenic processes they initiate. Notably, among the α-genus viruses, there is a solid consensus regarding their role as primary causal factors behind a range of cancers, including those of the cervix, genitalia, external genital regions, and oral cavity. Within this genus, the high-risk α-9 species, with HPV-16 as a prototypical member, is particularly notorious for its oncogenic potential, primarily driving infections that progress to cancerous states.

As an epithelium-specific, non-enveloped virus containing a circular double-stranded DNA genome, human papillomavirus (HPV) exclusively targets humans as its sole reservoir. Within the HPV genomic structure, distinct functional domains are present: (1) the non-coding region, which contains promoters, enhancers, and silencer sequences that play crucial roles in regulating viral DNA replication and transcriptional control; (2) the early gene region, comprising six key genes designated E1 through E7, which governs viral infection, replication, transcription, and translation processes (Chow, 2015; Ghoreshi et al., 2023); (3) the late gene region, consisting of the L1 and L2 genes, which are responsible for synthesizing the structural proteins essential for forming the viral capsid. The central proteins, E1 and E2, actively participate in replicating the viral genome, while L1 and L2 contribute to the assembly of virus particles. Additionally, the auxiliary proteins E4, E5, E6, and E7 orchestrate functions critical for accelerating cell proliferation, evading the immune system, and facilitating the release of new virions (Graham, 2017).

In benign tumors and low-grade precancerous lesions, the HPV genome predominantly exists in an episomal form. In contrast, in malignant tumors and high-grade precancerous lesions, it frequently integrates into the host cell genome, either as single or multiple copies (Woodman et al., 2007). This genomic incorporation primarily occurs following the early genes E6 and E7, which are often located near the E2 hinge domain. Disruption of E2 results in the inactivation of its open reading frame (ORF), leading to the loss of its negative feedback regulation over the transcription of the carcinogenic E6 and E7. This loss results in their aberrant overexpression and subsequent uncontrolled cellular proliferation (Munoz et al., 2003).

E6 and E7 play crucial roles in the processes of cellular immortalization by interacting with key cellular regulators such as p53 and pRb. This interaction disrupts normal cell cycle regulation, apoptosis, and chromosomal stability (Moody and Laimins, 2010). E6 and E7 also impair spindle checkpoint function during mitosis and induce abnormal centrosome numbers, which increases genomic instability and may lead to chromosomal rearrangements and copy number variations (Duensing et al., 2000). Additionally, E6 influences telomerase activity by targeting NFX1–91 for degradation through the E6-AP-mediated ubiquitin-proteasome pathway. This action results in decreased repression of the hTERT promoter and elevated telomerase activity (Xu et al., 2008). E7 further contributes to immune evasion by inactivating interferon regulatory factor 1 (IRF1), allowing the pathogen to evade immunological detection and thereby facilitating the onset and maintenance of a chronic disease state (Park et al., 2000). Furthermore, the integration of HPV into the host genome disrupts the regulatory mechanisms that govern host gene transcription, leading to the loss or inactivation of critical tumor suppressor genes, increased expression of host oncogenes, alterations in host chromosomal structures, and changes in host gene methylation patterns. Collectively, these factors significantly contribute to carcinogenesis (Filho et al., 2017; Hu et al., 2015; Ojesina et al., 2014; Parfenov et al., 2014; Rusan et al., 2015).

## 3 Current methods of treatment for HPV

Vaccination, as a primary preventive measure, stands out as the most effective long-term strategy to reduce the prevalence of high-risk HPV strains, with the goal of eradicating HPV-associated malignancies over time (Stanley et al., 2012). Currently, three types of prophylactic HPV vaccines have received global approval: the bivalent, quadrivalent, and nonavalent HPV vaccines, all of which utilize HPV virus-like particles (VLPs) as antigens. Despite the availability of these vaccines, their protective efficacy is limited for individuals who have previously been infected with HPV, and the cross-protection provided by type-specific neutralizing antibodies is notably restricted (Clifford et al., 2003; Schiller et al., 2012). Secondary prevention involves routine cervical cancer screening via Pap smears or HPV DNA testing, enabling early detection of precancerous lesions (Perkins et al., 2020).

For HPV positive cancer patients, therapeutic strategies include: surgical excision, LEEP/conization for cervical dysplasia (Wang et al., 2022); radiotherapy/chemotherapy, cisplatin-based regimens for advanced cervical cancer (Small et al., 2017); immunotherapy, PD-1 inhibitors like pembrolizumab for recurrent/metastatic cervical cancer (Allouch et al., 2020). For established HPV infections in patients with precancerous lesions, commonly utilized pharmacological agents include antimitotic compounds, caustic substances, interferon inducers, and polyphenols (Schiffman et al., 2016). Presently, the absence of clinically effective virus-specific therapeutic agents for eradicating HPV is palpable, and ongoing preclinical investigations into nucleic acid-based therapeutics such as RNA interference (RNAi), ribozymes, and antisense RNA have only achieved partial suppression of HPV gene expression, falling short of completely eliminating the virus. Consequently, there is an escalating imperative for the development of more potent and secure HPV precision therapies and the formulation of more efficacious virus-targeted pharmaceuticals (Khairuddin et al., 2014; Kumar et al., 2014; Malecka et al., 2014).

## 4 CRISPR/Cas system

Genome editing, the precise and targeted modification of genetic material in organisms, represents a monumental advancement in molecular biology. In recent years, the development of a range of DNA nuclease-based gene editing tools has led to significant progress. Beginning with the groundbreaking introduction of zinc-finger nucleases (ZFNs), advancing to the next generation of transcription activator-like effector nucleases (TALENs), and culminating with the clustered regularly interspaced short palindromic repeats/CRISPR-associated protein (CRISPR/Cas) systems, the precision, cost-effectiveness, and breadth of applications for gene editing have expanded dramatically.

Originally conceived as an adaptive immune response mechanism in prokaryotes, specifically bacteria and archaea, the CRISPR/Cas system serves as a critical defense against viral and bacteriophage incursions. The ability of CRISPR/Cas9 to bind and cleave DNA targets *in vitro* has led to its harnessing as a revolutionary tool for gene editing (Jinek et al., 2012). The CRISPR-Cas system is categorized into two main classes: Class I, which encompasses subtypes I, III, and IV, and Class II, which includes subtypes II, V, and VI (Makarova et al., 2017a, 2017b). Subsequent research by Feng Zhang’s group identified several ribonucleases, including Cpf1 (Cas12a), C2c1 (Cas12b), C2c2 (Cas13a), and C2c3 (Cas13c), and engineered mutant variants of Cas9 from Staphylococcus aureus (SaCas9) and Streptococcus pyogenes (SpCas9) that demonstrate enhanced precision in DNA targeting (Abudayyeh et al., 2017, 2016; Ran et al., 2015; Strecker et al., 2019; Zetsche et al., 2015).

Currently, the CRISPR/Cas system stands out as the predominantly employed instrument for gene editing. Its mechanism involves the recruitment of the Cas9 protein to specific genomic DNA targets, guided by a single-guide RNA (sgRNA), which results in a double-strand break (DSB) at the targeted site. The induction of this DSB activates the cell’s intrinsic DNA repair pathways, primarily involving homologous recombination (HR) and non-homologous end joining (NHEJ). The latter represents a precise repair mechanism that demands a homologous donor DNA sequence as a template for accurate gene correction or integration (Symington and Gautier, 2011). By utilizing CRISPR/Cas technology, scientists can edit DNA sequences with remarkable precision, facilitating microbial genome editing, optimizing plant and animal breeds, constructing animal models, and treating genetic disorders (Hsu et al., 2014; Ran, 2017).

## 5 CRISPR/Cas-based therapy for HPV

The advent of CRISPR/Cas genome editing has revolutionized the landscape of antiviral therapeutic strategies, positioning itself as a potent weapon in the battle against viral infections, marking substantial progress in addressing HIV, HBV, and HSV (Cisneros et al., 2022; OhAinle et al., 2018; Sakuma et al., 2016; Yin et al., 2021; Zhang et al., 2021; Zhen et al., 2015). This innovative antiviral strategy has heralded new vistas for the treatment of HPV. Contemporary pharmacotherapies primarily mitigate further HPV infections in hosts but fail to eradicate the virus already established in patients. Even after therapeutic interventions, the virus may persist in a quiescent or latent state, ready for reactivation. A critical factor driving HPV-induced carcinogenesis is the integration of viral DNA into the host cell’s genome, a process that proves challenging to reverse with conventional antiviral agents once integration has occurred.

Gene editing technology, however, can directly target and cut viral DNA, thereby precisely eliminating the virus. Presently, CRISPR/Cas applications for HPV predominantly focus on excising the E7 and E6 oncogenes. Furukawa et al. have edited HPV-specific cytotoxic T lymphocytes (HPV-rejT) using CRISPR technology, enabling these T cells to effectively attack cervical cancer cells without triggering immune system rejection in patients. This strategy not only eludes immune surveillance by CD8^+^ T lymphocytes and NK cells but also, by selecting for HLA-A24, ensures compatibility with a broad patient demographic (Furukawa et al., 2023). Gao et al. have crafted an HPV16 E7-specific CRISPR/Cas tool and evaluated its editing efficacy relative to ZFN and TALEN technologies. In their investigation utilizing a K14-HPV16 transgenic mouse model, characterized by HPV-driven spontaneous cervical cancer development, they discovered that effectively eliminating the E7 gene led to the restored expression of CDK2, E2F1, and RB, thereby triggering apoptotic pathways in tumor cells and effectively reversing the progression of cervical cancer (Gao et al., 2022). An HPV16 E7-specific sgRNA-guided CRISPR/Cas system could precisely disrupt HPV16-E7 DNA at targeted loci, inducing apoptosis in HPV-positive SiHa and Caski cells. The precise disruption of the E7 gene led to a substantial reduction in E7 oncoprotein expression, concomitantly stimulating the augmentation of pRb tumor suppressor protein levels (Hu et al., 2014).

**Figure 1:**
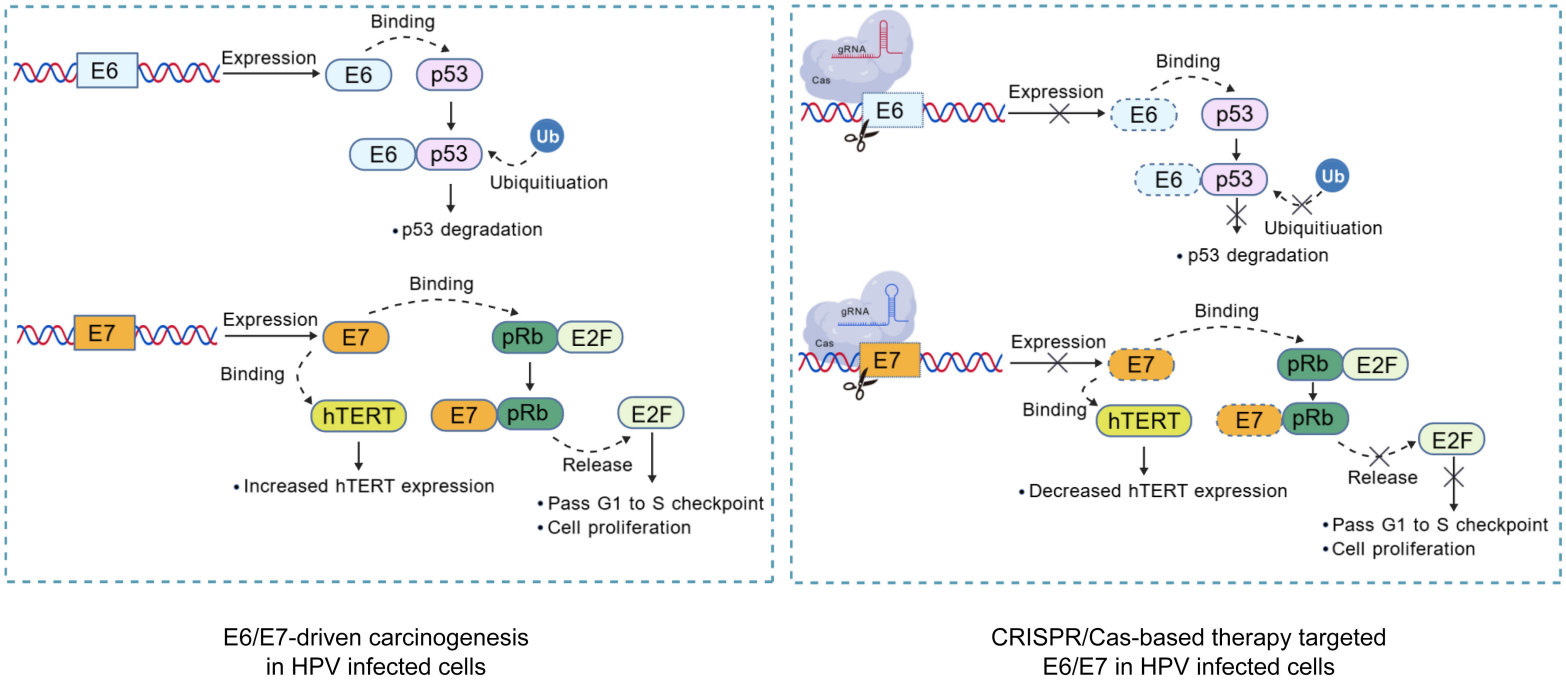
Mechanism of CRISPR/Cas targeted HPV oncogenes editing in Infected cells. Virus-specific gRNAs were designed to guide Cas in cleaving the HPV genome, disrupting the carcinogenic pathways. Highlighted pathways include the degradation of tumor suppressor p53 and pRb mediated by E6 and E7, respectively. The inactivation of pRb protein leads to the release of E2F transcription factors, resulting in cell cycle dysregulation, and E6 could activate hTERT activity. Created with BioGDP.com (Jiang et al., 2025).

Moreover, several investigations have developed CRISPR/Cas tools that utilize guide RNAs (gRNAs) to simultaneously target both the E6 and E7 genes (**Figure 1**). This dual-targeting strategy likely arises from the understanding that addressing a singular genomic locus might permit the viral genome to repair itself, potentially leading to mutations proximal to the cleavage site. By concurrently targeting both genes, these tools aim to comprehensively dismantle the HPV genome, thereby facilitating complete viral eradication. Zhen et al. engineered a CRISPR/Cas9-based editing system designed to simultaneously address the E7 and E6 genes and introduced it into SiHa cells. The findings revealed that the concurrent editing of the E6 and E7 genes, along with their respective promoters, via the CRISPR/Cas9 system, precipitated the accumulation of p53 and p21 proteins, significantly inhibiting the propagation of cervical carcinoma cell cultures under laboratory conditions. Following these *in vitro* results, the researchers conducted *in vivo* experiments by subcutaneously implanting the edited tumor cells into nude mice to establish an animal model of subcutaneous xenografts. The investigation noted a significant inhibition of neoplasm development in murine models engrafted with tumor cells that had previously undergone CRISPR/Cas9-mediated modification targeting the E6 and E7 genes (Zhen et al., 2014). Kennedy et al. developed a CRISPR/Cas editing tool capable of targeting and disabling the E6 and E7 genes within HeLa cells, inducing cellular cycle stasis and reinstating the expression profiles of p53 and pRb (Kennedy et al., 2014). **Table 1** summarizes the critical studies utilizing CRISPR/Cas9 technology to target specific gene sites in HPV infection research.

**Table 1:** Summary of selected studies of CRISPR/Cas9-based antiviral studies on targeting HPV.

Target virus	Type of gene editor	CRISPR targets	Efficacy	Delivery system	Ref.
HPV16	Cas9	E6	Edited T cells attack cervical cancer cells without immune rejection; HLA-A24 compatibility enables broad patient application	Plasmid stransfection	Furukawa et al., 2023
HPV16	Cas9	E7	Restored CDK2/E2F1/RB expression; induced apoptosis; Reversed cervical cancer progression in K14-HPV16 mice	Plasmid stransfection	Gao et al., 2022
HPV16	Cas9	E7	Reduced E7 oncoprotein; Increased pRb levels; Induced apoptosis in SiHa/Caski cells	Plasmid stransfection	Hu et al., 2014
HPV16	Cas9	E6, E7	Accumulated p53/p21; inhibited cervical cancer cell proliferation; Suppressed tumor growth in nude mice	Plasmids and lipofectamine delivery	Zhen et al., 2014
HPV18	Cas9	E6, E7	Induced cell cycle arrest; Restored p53/pRb expression in HeLa cells	Lentivirus delivery	Kennedy et al., 2014
HPV18	Cas9	E6	Reversed malignant phenotypes; Elevated p53 levels in HeLa cells	AAV delivery	Noroozi et al., 2022
HPV18	Cas9	E6	Mutated E6 sequences; Enhanced apoptosis; Suppressed tumor growth in mice without toxicity	AAV delivery	Yoshiba et al., 2019
HPV16, 18	Cas9	E6	Reduced viability in HeLa/SiHa/Caski cells	AdV delivery	Ehrke-Schulz et al., 2020
HPV16, 18	WTCas9,FokI dCas9	E6, E7	Achieved tumor eradication and 100% survival *in vivo*; Addressed serum stability/cytotoxicity challenges	PEGylated liposomes delivery	Jubair et al., 2019
HPV16	Cas9	E6, E7	pH-sensitive liposomes enabled tumor-specific delivery; Suppressed tumor growth without toxicity	Cationic liposomes delivery	Zhen et al., 2020a
HPV18	Cas9	E6, E7	Restored p53/pRB pathway; Induced apoptosis in cervical cancer cells	DOTAP liposomes delivery	Li et al., 2021
HPV16	Cas9	E6, E7	pH-responsive NPs showed high transfection, low cytotoxicity; Significant antitumor activity in xenografts	ACD-PEI nanoparticles delivery	Ling et al., 2023
HPV16	Cas9	E7	Suppressed tumor proliferation; Restored benign epithelial characteristics;	PBAE546 nanoparticles delivery	Xiong et al., 2021
HPV16	Cas9	E6, E7, E5	Suppressed oncogene expression; Reduced tumor proliferation in C57BL/6 mice	LL-37 antimicrobial peptide delivery	Khairkhah et al., 2023
HPV16	Cas9	E6, E7	Enhanced anti-PD-1 efficacy; Promoted CD8^+^ T-cell infiltration; Increased IFN-γ/TNF-α/IL-12; reduced MDSCs/Tregs	Liposomes delivery	Zhen et al., 2023

## 6 CRISPR/Cas delivery for HPV therapy

**Figure 2:**
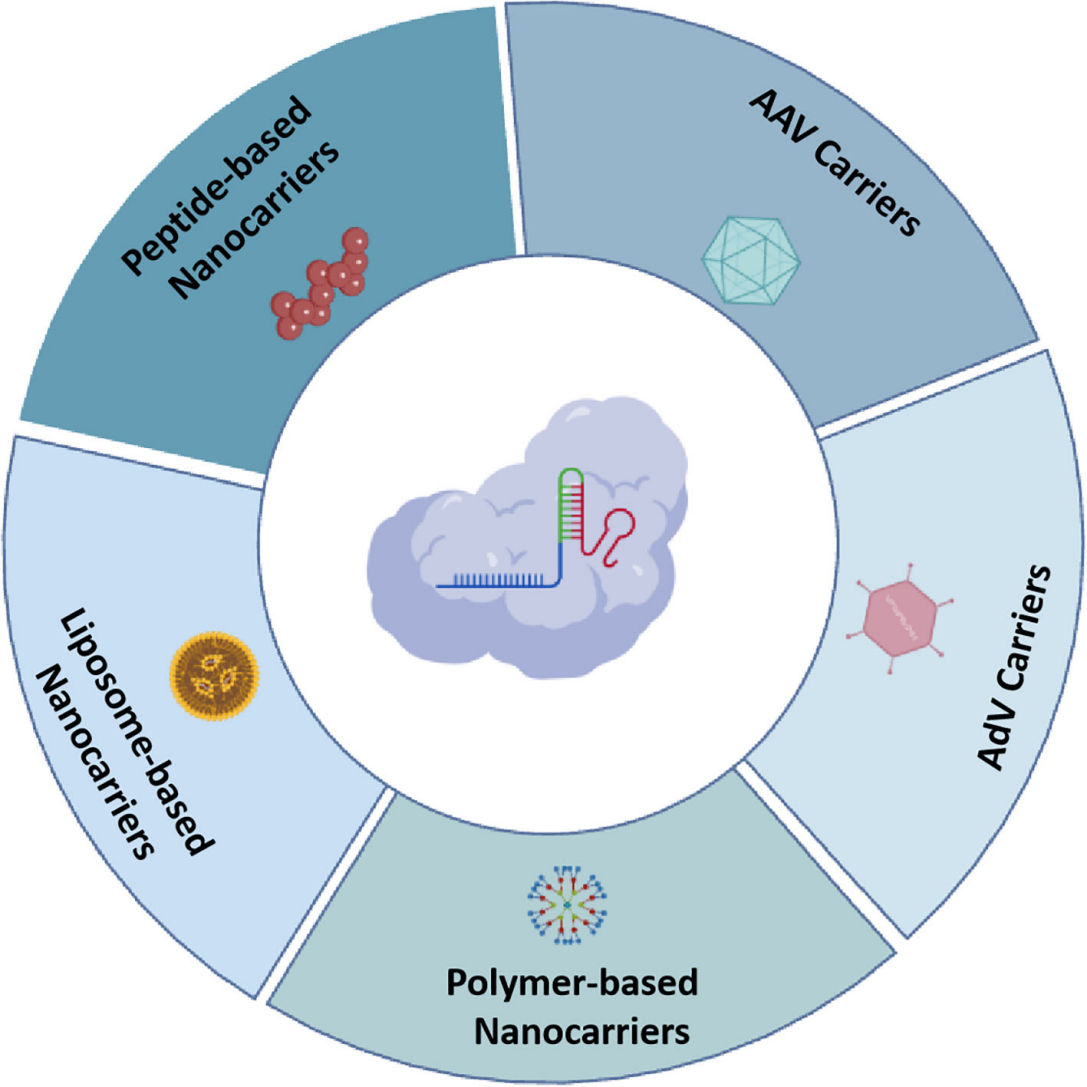
Overview of various CRISPR/Cas carrieres in HPV genome editing therapy, include: AAV carriers, AdV carriers, liposome-based nanocarriers, polymer-based nanocarriers, and peptide-based nanocarriers. Created with BioGDP.com (Jiang et al., 2025).

A significant challenge in deploying CRISPR/Cas editing technologies for therapeutic interventions in human diseases is the absence of effective delivery mechanisms that can precisely target specific cells and organs *in vivo*. Dr. Jennifer Doudna, a Nobel Laureate, has stated that, delivery remains perhaps the most significant bottlenecks in somatic cell gene therapy using gene editing. Currently, CRISPR gene editing tools are primarily delivered into cells through three principal modalities: plasmid DNA (pDNA), mRNA, and ribonucleoprotein complexes (RNP), utilizing either viral or non-viral vectors to facilitate targeted gene modification (Lin et al., 2022). Early research efforts primarily focused on utilizing plasmid vectors as the means to deliver the CRISPR/Cas system into targeted cells. This approach ensures stable expression and notable translational efficiency; however, its prolonged presence within cells poses potential risks, such as genomic integration, elicitation of immune response activation, and unintended off-target effects. Additionally, delivering the Cas gene via plasmids requires transcriptional and translational processes to achieve gene editing. In contrast, administering the CRISPR system in the form of mRNA or RNPs offers more immediate efficacy and greater controllability (Lin et al., 2022; Yan et al., 2021) (**Figure 2**).

### 6.1 Viral carriers

Over eons, viruses have refined their capabilities through natural evolution, enabling them to efficiently infiltrate diverse human tissue types and cellular environments, and viral particles can protect the encapsulated payload from degradation within the body. Upon entry into the host cell, these recombinant viruses facilitate the efficient expression of exogenous genes. Upon entry into the host cell, these recombinant viruses facilitate the efficient expression of exogenous genes. To date, viral vectors remain the preeminent method for delivering CRISPR/Cas systems to mammalian cells *in vivo*, significantly enhancing the precision and efficiency of gene editing. Frequently utilized viral carriers for the conveyance of CRISPR/Cas include adenovirus (AdV), adeno-associated virus (AAV), retrovirus, and lentivirus (Miron-Barroso et al., 2021).

#### 6.1.1 AAV carriers

Presently, more than fifty percent of gene therapy vehicles applied in clinical investigations are of viral origin, among which AAV stands out as the most prevalently employed. Extensive foundational and clinical research has demonstrated that AAV has high safety and delivery efficiency, making it broadly applied in the field of gene therapy (Lau and Suh, 2017; Wang et al., 2020). AAV is a non-pathogenic parvovirus found in various vertebrates, including humans and non-human primates (NHP), although wild-type AAV may integrate into specific genomic sites, recombinant AAV (rAAV) vectors predominantly persist as episomes in host cells, resulting in a significantly reduced risk of insertional mutagenesis compared to integrating viral vectors (Naso et al., 2017). The diversity of AAV serotypes, such as AAV2 and AAV5, facilitates cell-specific targeting, thereby enabling precision therapy. Crucially, AAV elicits minimal immune response in hosts, an attribute essential for sustained therapeutic interventions. Importantly, cells transduced by AAV maintain expression of therapeutic genes without precipitating cellular demise or uncontrolled proliferation, affirming its role as a stable and secure therapeutic vector.

Noroozi et al. employed AAV to deliver CRISPR/Cas9, achieving specific cleavage of the HPV E6 gene in HeLa cells, which reversed malignant phenotypes and augmented p53 protein levels (Noroozi et al., 2022). Kennedy et al. demonstrated that a lentivirus vector encoding HPV18 E6 and E7 gene-specific CRISPR/Cas9 triggered programmed cell death in cervical cancer cell (Kennedy et al., 2014). In similar research, a Lentivirus CRISPR/Cas9 system directed against the HPV18 E6 or E7 genes efficiently repressed the expression of these carcinogenic factors in HeLa cells, inducing a halt in the cell cycle and diminished cell multiplication (Wang et al., 2018). Yoshiba et al. combined a cervical cancer cell line expressing recombinant Cas9 with an AAV-sgE6 vector and observed *in vitro* that E6 genomic sequences were mutated, expression diminished, p53 expression increased, apoptosis was enhanced, and cellular proliferation was restrained in a concentration-related fashion. Moreover, *in vivo* experiments demonstrated that post-injection of AAV-sgE6, the growth of subcutaneous tumors in tumor-bearing mice was significantly curtailed without observable adverse effects (Yoshiba et al., 2019). Noroozi’s findings indicated the efficacy of utilizing AAV vector for CRISPR/Cas9 delivery in targeting the HPV-E6 gene within HeLa cells, thereby enhancing tumor cell apoptosis and elevating p53 expression levels (Noroozi et al., 2022).

#### 6.1.2 AdV carriers

AdV is characterized by its non-enveloped structure, featuring an icosahedral nucleocapsid and a double-stranded DNA genome that spans approximately 26–45 kbs. AdVs, with a diameter of 90–100 nm, can accommodate up to 38 kbs of exogenous DNA, a capacity significantly greater than that of AAV. Distinct from AAV, the AdV genome remains episomal within the host cell, not integrate into the host genome. This transient expression is advantageous for CRISPR/Cas delivery, as it minimizes the potential for off-target genetic alterations.

Despite these attributes, adenoviral vectors exhibit limited tropism toward tissues commonly affected by HPV, which constrains their utility in treating HPV-related conditions (Alonso-Padilla et al., 2016). Eric et al. engineered a CRISPR/Cas9-HCAdV system that enables sustained or inducible expression of Cas9/gRNA targeting the HPV18 E6 gene, successfully inducing double-strand breaks (DSBs) in HeLa cells (Ehrke-Schulz et al., 2017). They effectively delivered the HPV type-specific CRISPR-HCAdV to not just HeLa, SiHa, and CaSki cells but also to HPV-negative cancer cells. Following delivery, CRISPR/Cas9 expression resulted in decreased viability in HPV-positive cervical cancer cell lines, while leaving HPV-negative cells unharmed (Ehrke-Schulz et al., 2020).

However, the relatively high immunogenicity of AdVs poses a significant challenge, particularly *in vivo*, where it can provoke allergic reactions. From the second injection onward, the efficacy of the delivery may decrease due to immune responses. Consequently, while AdVs are advantageous for certain applications due to their large payload capacity and transient expression characteristics, their clinical application is complicated by potential immunogenic responses, necessitating careful management in therapeutic settings.

### 6.2 Nanocarriers

Despite the notable transfection efficiency exhibited by viral vectors, their clinical adoption is impeded by immune-mediated rejection and limitations in cargo capacity. The presence of pre-existing immunity against wild-type viral vectors in certain populations, coupled with the restricted packaging capabilities of AAVs, has ignited a wave of research focused on the engineering of non-viral delivery systems, aiming to attain substantial progress in this domain (Li et al., 2018; Van Hees et al., 2022). Nanocarriers engineered from an array of natural or synthetic polymers, lipid constituents, proteinaceous macromolecules, and inorganic entities, are stratified into four principal categories: liposomal carriers, polymeric carriers, inorganic carriers, and biologically-derived carriers. Concomitant with progressive innovations in material science and manufacturing techniques, those nano-carriers, which are cost-effective, straightforward to synthesize, and amenable to purification, have been distinguished by their elevated transfection efficacy and minimal immunogenicity. Consequently, they have ascended as preeminent vectors for the administration of CRISPR/Cas genome-editing apparatuses, specifically targeting the HPV genome.

#### 6.2.1 Liposome-based nanocarriers

Liposomes, composed of cholesterol and non-toxic phospholipids, create robust spherical vesicles capable of merging with cell membranes or being engulfed through endocytosis, thus enabling the efficient intracellular delivery of CRISPR elements. Owing to their multifunctional architecture, facile surface modifiability, biodegradability, and biocompatibility, liposomes are heralded as a formidable system for gene therapy delivery (Guimaraes et al., 2021).

Commercial liposomal carriers, such as Lipofectamine™, TurboFect™, and Stemfect™, have demonstrated efficacy in transporting the CRISPR/Cas9 system. The modification with polyethylene glycol (PEG) enhances the *in vivo* stability and prolongs the circulation time of liposomes (Milla et al., 2012). Jubair et al. utilized PEGylated liposomes for the systemic administration of CRISPR/Cas9 targeting the HPV16/18 E6/E7 genes *in vivo*, achieving tumor eradication and complete survival in treated models. This strategy addresses several challenges associated with the systemic delivery of CRISPR/Cas9, including serum stability, cytotoxicity, and cellular uptake of the therapeutic payload (Jubair et al., 2019). Positively charged liposomes can form conjugates with negatively charged CRISPR components through electrostatic attraction, effectively encapsulating and delivering Cas proteins and gRNA into target cells (Sousa et al., 2022). Zhen et al. developed long-circulating, pH-sensitive cationic liposomal complexes that exhibit high cell-targeting and gene knockout efficiencies, selectively delivering encapsulated HPV16 E6/E7 CRISPR/Cas9 complexes to tumor tissues and significantly suppressing tumor growth without notable toxicity *in vivo* (Zhen et al., 2020a). Subsequent investigations by their group revealed that liposomes loaded with CRISPR/Cas9 effectively knock out HPV16 E6/E7, initiating autophagy and stimulating immune responses associated with cellular death through the release of danger-associated molecular patterns. This process promotes the accumulation of CD8^+^ T lymphocytes within neoplastic tissues and enhances the production of pro-inflammatory mediators, including IFN-γ, TNF-α, and IL-12, while reducing myeloid-derived suppressor cells (MDSCs) and regulatory T cells (Tregs) (Zhen et al., 2023). Li et al. developed a CRISPR/Cas9 assembly encapsulated within the hydrophobic bilayer of amphiphilic cationic liposomes composed of DOTAP. This strategy precisely targeted and removed approximately 563 base pairs from the HPV18 E6 and E7 oncogenes, successfully eradicating the viral DNA sequences embedded within the host’s genomic framework. Consequently, this intervention restored the p53 and pRB tumor-suppressive signaling pathways, leading to the induction of apoptotic cell death in cervical cancer cells (Li et al., 2021).

#### 6.2.2 Polymer-based nanocarriers

Organic polymers possess the capability to form intricate molecular assemblies with the CRISPR/Cas complex, which can be functionalized to package various components to enhance cellular or tissue specificity, intracellular uptake, and endosomal escape (Dilliard and Siegwart, 2023). Compared to liposomes, polymer carriers present a broader spectrum of chemical variability and functional capabilities, offering enhanced versatility in structural customization, and can directly interact with CRISPR/Cas complexes to improve their delivery characteristics (Duan et al., 2021).

Polyethyleneimine (PEI) is a hydrophilic cationic polymer available in both linear and branched configurations with variable molecular weights. It is highly effective at complexing and condensing negatively charged DNA through electrostatic interactions and possesses inherent pH buffering capabilities that facilitate endosomal escape. Ling et al. engineered pH-responsive nanoparticles (NPs) by combining acetylated cyclo-oligosaccharides (ACD) with low molecular weight PEI to simultaneously transport Cas9 mRNA and gRNAs aimed at the E7 and E6 oncogenes. Their findings revealed that, *in vitro*, ACD-stabilized NPs demonstrated substantial transfection efficacy alongside minimal cytotoxic effects in HeLa cervical cancer cells. Furthermore, they successfully manipulated specific genetic loci with minimal off-target effects was achieved in HeLa cells. In HeLa xenograft mice, treatment with E6/ACD NPs or E7/ACD NPs effectively edited target cancer genes and demonstrated significant antitumor activity (Ling et al., 2023).

Xiong et al. developed NPs comprised of the positively charged polymer PBAE546 in complex with a CRISPR/Cas9 plasmid targeting HPV16 E7. These NPs significantly inhibited the proliferation of xenografted cervical carcinoma cell lines in nude mouse models and restored the normal characteristics of cervical epithelial tissue in HPV16 transgenic mice, demonstrating low toxicity and high biosafety (Xiong et al., 2021).

Lao et al. designed self-assembling micelles composed of PPO-NMe3 in conjunction with the amphiphilic polymer Pluronic F127 for the delivery of CRISPR/Cas9 plasmids targeting HPV18 E7. These highly charged polycationic micelles effectively protected the plasmid from degradation, resulting in enhanced transgene expression. The Cas9-mediated knockout of E7 significantly reduced the oncogenic activity induced by HPV in both *in vitro* and *in vivo* settings (Lao et al., 2018).

#### 6.2.3 Peptide-based nanocarriers

Numerous studies have demonstrated that peptide complexes can effectively guide CRISPR/Cas tools through the cellular membranes and into the nucleus to achieve gene editing (Zhang et al., 2024). As an nascent drug delivery system, peptides exhibit high biological activity, their side chains are versatile scaffolds for various active functional groups such as carboxyl, hydroxyl, amino, and thiol, enabling a spectrum of chemical modifications to amplify the efficacy of drug delivery systems (Dissanayake et al., 2017). Investigations into peptide-mediated transport of macromolecules into cells have predominantly centered on CPPs, exemplified by the TAT sequence from HIV-1 and the HA2 segment from the influenza virus, an archetypal amphipathic peptide. HA2 is recognized for its role in mediating the disturbance of endosomal membrane integrity and the release of their cargoes into the cytoplasm through a pH-sensitive conformational shift (Noguchi et al., 2010).

To improve CRISPR/Cas delivery in cervical cancer treatment, Khairkhah et al. utilized the LL-37 antimicrobial peptide (AMP) as CPPs carrier. Their studies demonstrated that the tailored CRISPR/Cas9 system adeptly and selectively suppressed the expression of HPV16 E7, E6, and E5 genes both *in vitro* and within living organisms, consequently diminishing tumor proliferation in the C57BL/6 mouse model (Khairkhah et al., 2023).

However, the use of non-viral vectors for CRISPR/Cas delivery still faces two significant challenges. First, compared to viral vectors, the delivery efficiency of non-viral vectors remains lower. This is primarily due to the need for non-viral delivery of CRISPR/Cas needs to overcome multiple barriers, including both extracellular and intracellular obstacles (Mitchell et al., 2021; Nelson and Gersbach, 2016). Extracellular challenges include unintended interactions with serum proteins, phagocytosis by immune cells, and the impermeable junctions of vascular epithelial cells, which obstruct NPs penetration and targeting. Intracellularly, barriers consist of the degradation of NPs within endosomes and lysosomes, the complexities involved in releasing the CRISPR/Cas components into the cytoplasm, and the subsequent requirement for these components to enter the nucleus to function effectively. Second, achieving precise genome editing through the targeted delivery of the CRISPR/Cas system using non-viral vectors remains a formidable challenge (Tasset et al., 2022).

## 7 Combination therapy strategies

Recent progress in the field of immunotherapy, particularly with ICIs, have been integrated into clinical guidelines for the management of cervical cancer in the context of viral infections. However, the efficacy of ICIs as monotherapy for recurrent or metastatic cervical cancer remains suboptimal, with low response rates (Awadasseid et al., 2023). Consequently, researches has explored combining CRISPR/Cas therapy targeting HPV E6/E7 with ICIs to enhance therapeutic outcomes through synergistic effects. One promising approach demonstrated by Zhen et al. involved the use of liposomes to deliver gRNA targeting HPV16 E6/E7, which not only initiated anti-tumor immune responses but also augmented the effectiveness of anti-PD-1 treatment in a hu‐PBL‐SCID mice model, CRISPR/Cas9 targeting of HPV E6/E7 reactivates p53/pRb tumor suppression, triggering apoptotic death and blocking cancer progression, HPV knockout triggers autophagy and releases DAMPs, activating dendritic cells and CD8^+^ T cells, reversing immunosuppressive, and enhanced antitumor immunity via increased PD-1 blockade efficacy, promoting immune memory and sustained tumor regression (Zhen et al., 2023). This tactic capitalizes on the elevated expression of PD-1 on immune cells, a critical pathway through which tumors escape immune surveillance. By employing CRISPR/Cas tools to knock down or knock out PD-1, this approach effectively prevents tumor immune escape. In humanized SCID mice xenografted with SiHa cells, the combined treatment using gRNA-HPV16 E6/E7 and gRNA-PD-1 substantially boosted survival rates and curbed tumor progression. Moreover, this combination therapy augmented the infiltration of dendritic cells and CD4^+^ and CD8^+^ T lymphocytes, concurrently diminishing the transcription of immunosuppressive genes, thereby transforming the tumor microenvironment from one characterized by immunosuppression to an activated state (Zhen et al., 2020b). These findings underscore the potential of combining targeted HPV CRISPR/Cas therapy with ICIs in clinical settings for treating cervical cancer.

For individuals afflicted with recurrent or disseminated cervical cancer, where curative surgery is not feasible, chemotherapy remains a primary treatment option. However, the efficacy of chemotherapeutic monotherapy is often limited, resulting in low response rates and short remission periods. The evolution of CRISPR/Cas technology heralds novel prospects for augmenting the therapy of HPV-related tumors, particularly when used in combination with chemotherapy. For instance, in a nude mouse model xenografted with HeLa cells, delivering a CRISPR/Cas9 engineered plasmid directed at HPV18 E6/E7 in conjunction with DOC dramatically enhanced the cytotoxicity toward cervical cancer cells by overcoming drug resistance via p53/pRB-dependent sensitization, leading to an increased apoptotic response, which suggests an improved management of resistance, simultaneous knockout of HPV-18 E6/E7 oncogenes disrupts viral oncoprotein expression, reversing chemoresistance, cationic liposomes ensure precise tumor localization, while AIE fluorophores enable real-time tracking, minimizing off-target effects (Li et al., 2021). Further, Zhen et al. illustrated that harnessing HPV16 E6/E7 with CRISPR/Cas9 could potently complement cisplatin in combating HPV16-positive cervical cancer within a xenograft framework, thereby enhancing patient outlook (Zhen et al., 2016).

## 8 Conclusion and future perspective

As a revolutionary gene editing tool, CRISPR offers novel perspectives and promising avenues for the treatment of HPV. By precisely targeting and cleaving key HPV genes such as E6 and E7, CRISPR not only has the potential to eradicate the virus but also to prevent the cellular carcinogenesis initiated by HPV, thereby reducing the prevalence of HPV-associated abnormalities and cancers.

Currently, research on CRISPR/Cas tools for HPV treatment remains in the laboratory stage, with only one clinical trial assessing the security and effectiveness of CRISPR/Cas9-HPV E6/E7 and TALEN-HPV E6/E7 in managing persistent HPV infections and HPV-related CIN I during Phase I clinical stage (NCT03057912) (Hu et al., 2014). CRISPR/Cas gene editing confronts critical translational hurdles, notably off-target editing events, delivery constraints, and immunogenicity. Off-target mutations, arising from Cas nuclease promiscuity or partial homology, pose significant genotoxicity risks. Mitigation leverages high-fidelity engineered nucleases (e.g., HiFi Cas9, Cas12a variants), rational design of gRNA, and comprehensive off-target profiling via CIRCLE-seq or GUIDE-seq. Delivery bottlenecks stem from biological barriers, macromolecular complex size, and nuclease stability. While viral vectors offer efficiency, they face cargo limitations and insertional mutagenesis concerns; non-viral strategies (lipid nanoparticles, polymer-based carriers, electroporation) grapple with potency and cell-type specificity. Targeted delivery necessitates advanced ligand engineering and tissue-specific formulations. Immunogenicity presents dual challenges: pre-existing humoral/cellular immunity to microbial Cas orthologs and vector-triggered immune responses, compromising efficacy and safety. Countermeasures include exploiting rare orthologs (e.g., Cas12f), structure-guided deimmunization of Cas proteins via epitope deletion/masking, and transient mRNA/protein delivery to minimize antigen persistence. Overcoming these challenges through precision editors (base/prime editing), next-generation delivery platforms, and immunomodulatory strategies is paramount for realizing CRISPR’s therapeutic promise.

Despite encountering obstacles such as suboptimal editing efficiency, notable immunogenicity, and the absence of a reliable and efficacious delivery system, the prospective impact of CRISPR/Cas technology in combating HPV remains revolutionary. Future research endeavors should be directed towards refining the design of the CRISPR/Cas system to enhance both its safety and efficacy. Additionally, there is a critical need to explore synergistic integrations with other therapeutic modalities, including vaccines, immunotherapies, and antiviral drugs, to forge a more holistic and potent treatment strategy.

## References

[B1] Abudayyeh O. O. Gootenberg J. S. Essletzbichler P. Han S. Joung J. Belanto J. J.. (2017). RNA targeting with CRISPR-Cas13. Nature. 550, 280–284. doi: 10.1038/nature24049, PMID: 28976959 PMC5706658

[B2] Abudayyeh O. O. Gootenberg J. S. Konermann S. Joung J. Slaymaker I. M. Cox D. B.. (2016). C2c2 is a single-component programmable RNA-guided RNA-targeting CRISPR effector. Science 353, aaf5573. doi: 10.1126/science.aaf5573, PMID: 27256883 PMC5127784

[B3] Alemany L. Cubilla A. Halec G. Kasamatsu E. Quiros B. Masferrer E.. (2016). Role of human papillomavirus in penile carcinomas worldwide. Eur. Urol. 69, 953–961. doi: 10.1016/j.eururo.2015.12.007, PMID: 26762611

[B4] Alemany L. Saunier M. Alvarado-Cabrero I. Quiros B. Salmeron J. Shin H. R.. (2015). Human papillomavirus DNA prevalence and type distribution in anal carcinomas worldwide. Int. J. Cancer. 136, 98–107. doi: 10.1002/ijc.28963, PMID: 24817381 PMC4270372

[B5] Allouch S. Malki A. Allouch A. Gupta I. Vranic S. Al Moustafa A. E. (2020). High-risk HPV oncoproteins and PD-1/PD-L1 interplay in human cervical cancer: recent evidence and future directions. Front. Oncol. 10. doi: 10.3389/fonc.2020.00914, PMID: 32695664 PMC7338567

[B6] Alonso-Padilla J. Papp T. Kajan G. L. Benko M. Havenga M. Lemckert A.. (2016). Development of novel adenoviral vectors to overcome challenges observed with HAdV-5-based constructs. Mol. Ther. 24, 6–16. doi: 10.1080/21645515.2017.1419108, PMID: 26478249 PMC4754553

[B7] Awadasseid A. Zhou Y. Zhang K. Tian K. Wu Y. Zhang W. (2023). Current studies and future promises of PD-1 signal inhibitors in cervical cancer therapy. BioMed. Pharmacother. 157, 114057. doi: 10.1016/j.biopha.2022.114057, PMID: 36463828

[B8] Bruni L. Albero G. Serrano B. Mena M. Collado J. J. Gómez D.. (2023). .ICO/IARC Information Centre on HPV and Cancer (HPV InformationCentre). Human Papillomavirus and Related Diseases in the World. Summary Report. Available online at: https://hpvcentre.net/statistics/reports/XWX.pdf

[B9] Burk R. D. Harari A. Chen Z. (2013). Human papillomavirus genome variants. Virology. 445, 232–243. doi: 10.1016/j.virol.2013.07.018, PMID: 23998342 PMC3979972

[B10] Chow L. T. (2015). Model systems to study the life cycle of human papillomaviruses and HPV-associated cancers. Virol. Sin. 30, 92–100. doi: 10.1007/s12250-015-3600-9, PMID: 25924993 PMC8200882

[B11] Cisneros W. J. Cornish D. Hultquist J. F. (2022). Application of CRISPR-cas9 gene editing for HIV host factor discovery and validation. Pathogens. 11, 891. doi: 10.3390/pathogens11080891, PMID: 36015010 PMC9415735

[B12] Clifford G. M. Smith J. S. Plummer M. Munoz N. Franceschi S. (2003). Human papillomavirus types in invasive cervical cancer worldwide: a meta-analysis. Br. J. Cancer. 88, 63–73. doi: 10.1038/sj.bjc.6600688, PMID: 12556961 PMC2376782

[B13] Dilliard S. A. Siegwart D. J. (2023). Passive, active and endogenous organ-targeted lipid and polymer nanoparticles for delivery of genetic drugs. Nat. Rev. Mater 8, 282–300. doi: 10.1038/s41578-022-00529-7, PMID: 36691401 PMC9850348

[B14] Dissanayake S. Denny W. A. Gamage S. Sarojini V. (2017). Recent developments in anticancer drug delivery using cell penetrating and tumor targeting peptides. J. Control Release. 250, 62–76. doi: 10.1016/j.jconrel.2017.02.006, PMID: 28167286

[B15] Doorbar J. Quint W. Banks L. Bravo I. G. Stoler M. Broker T. R.. (2012). The biology and life-cycle of human papillomaviruse. Vaccine 30 Suppl 5, F55–F70. doi: 10.1016/j.vaccine.2012.06.083, PMID: 23199966

[B16] Duan L. Ouyang K. Xu X. Xu L. Wen C. Zhou X.. (2021). Nanoparticle delivery of CRISPR/cas9 for genome editing. Front. Genet. 12. doi: 10.3389/fgene.2021.673286, PMID: 34054927 PMC8149999

[B17] Duensing S. Lee L. Y. Duensing A. Basile J. Piboonniyom S. Gonzalez S.. (2000). The human papillomavirus type 16 E6 and E7 oncoproteins cooperate to induce mitotic defects and genomic instability by uncoupling centrosome duplication from the cell division cycle. Proc. Natl. Acad. Sci. U.S.A. 97, 10002–10007. doi: 10.1073/pnas.170093297, PMID: 10944189 PMC27652

[B18] Ehrke-Schulz E. Heinemann S. Schulte L. Schiwon M. Ehrhardt A. (2020). Adenoviral vectors armed with PAPILLOMAVIRUs oncogene specific CRISPR/Cas9 kill human-papillomavirus-induced cervical cancer cells. Cancers (Basel). 12, 1934. doi: 10.3390/cancers12071934, PMID: 32708897 PMC7409089

[B19] Ehrke-Schulz E. Schiwon M. Leitner T. David S. Bergmann T. Liu J.. (2017). CRISPR/Cas9 delivery with one single adenoviral vector devoid of all viral genes. Sci. Rep. 7, 17113. doi: 10.1038/s41598-017-17180-w, PMID: 29215041 PMC5719366

[B20] Faber M. T. Sand F. L. Albieri V. Norrild B. Kjaer S. K. Verdoodt F. (2017). Prevalence and type distribution of human papillomavirus in squamous cell carcinoma and intraepithelial neoplasia of the vulva. Int. J. Cancer. 141, 1161–1169. doi: 10.1002/ijc.30821, PMID: 28577297

[B21] Filho S. M. A. Bertoni N. Brant A. C. Vidal J. Felix S. P. Cavalcanti S. M. B.. (2017). Methylation at 3’LCR of HPV16 can be affected by patient age and disruption of E1 or E2 genes. Virus Res. 232, 48–53. doi: 10.1016/j.virusres.2017.01.022, PMID: 28143725

[B22] Furukawa Y. Ishii M. Ando J. Ikeda K. Igarashi K. J. Kinoshita S.. (2023). iPSC-derived hypoimmunogenic tissue resident memory T cells mediate robust anti-tumor activity against cervical cancer. Cell Rep. Med. 4, 101327. doi: 10.1016/j.xcrm.2023.101327, PMID: 38091985 PMC10772465

[B23] Gao C. Wu P. Yu L. Liu L. Liu H. Tan X.. (2022). The application of CRISPR/Cas9 system in cervical carcinogenesis. Cancer Gene Ther. 29, 466–474. doi: 10.1038/s41417-021-00366-w, PMID: 34349239 PMC9113934

[B24] Ghoreshi Z. A. Molaei H. R. Arefinia N. (2023). The role of DNA viruses in human cancer. Cancer Inform 22, 11769351231154186. doi: 10.1177/11769351231154186, PMID: 37363356 PMC10286548

[B25] Graham S. V. (2017). The human papillomavirus replication cycle, and its links to cancer progression: a comprehensive review. Clin. Sci. (Lond) 131, 2201–2221. doi: 10.1042/CS20160786, PMID: 28798073

[B26] Guimaraes D. Cavaco-Paulo A. Nogueira E. (2021). Design of liposomes as drug delivery system for therapeutic applications. Int. J. Pharm. 601, 120571. doi: 10.1016/j.ijpharm.2021.120571, PMID: 33812967

[B27] Haedicke J. Iftner T. (2013). Human papillomaviruses and cancer. Radiother Oncol. 108, 397–402. doi: 10.1016/j.radonc.2013.06.004, PMID: 23830197

[B28] Hsu P. D. Lander E. S. Zhang F. (2014). Development and applications of CRISPR-Cas9 for genome engineering. Cell. 157, 1262–1278. doi: 10.1016/j.cell.2014.05.010, PMID: 24906146 PMC4343198

[B29] Hu Z. Yu L. Zhu D. Ding W. Wang X. Zhang C.. (2014). Disruption of HPV16-E7 by CRISPR/Cas system induces apoptosis and growth inhibition in HPV16 positive human cervical cancer cells. BioMed. Res. Int. 2014, 612823. doi: 10.1155/2014/612823, PMID: 25136604 PMC4127252

[B30] Hu Z. Zhu D. Wang W. Li W. Jia W. Zeng X.. (2015). Genome-wide profiling of HPV integration in cervical cancer identifies clustered genomic hot spots and a potential microhomology-mediated integration mechanism. Nat. Genet. 47, 158–163. doi: 10.1038/ng.3178, PMID: 25581428

[B31] Jiang S. Li H. Zhang L. Mu W. Zhang Y. Chen T.. (2025). Generic Diagramming Platform (GDP): a comprehensive database of high-quality biomedical graphics. Nucleic Acids Res. 53, D1670–D1676. doi: 10.1093/nar/gkae973, PMID: 39470721 PMC11701665

[B32] Jinek M. Chylinski K. Fonfara I. Hauer M. Doudna J. A. Charpentier E. (2012). A programmable dual-RNA-guided DNA endonuclease in adaptive bacterial immunity. Science. 337, 816–821. doi: 10.1126/science.1225829, PMID: 22745249 PMC6286148

[B33] Jubair L. Fallaha S. McMillan N. A. J. (2019). Systemic delivery of CRISPR/cas9 targeting HPV oncogenes is effective at eliminating established tumors. Mol. Ther. 27, 2091–2099. doi: 10.1016/j.ymthe.2019.08.012, PMID: 31537455 PMC6904748

[B34] Kennedy E. M. Kornepati A. V. Goldstein M. Bogerd H. P. Poling B. C. Whisnant A. W.. (2014). Inactivation of the human papillomavirus E6 or E7 gene in cervical carcinoma cells by using a bacterial CRISPR/Cas RNA-guided endonuclease. J. Virol. 88, 11965–11972. doi: 10.1128/JVI.01879-14, PMID: 25100830 PMC4178730

[B35] Khairkhah N. Bolhassani A. Rajaei F. Najafipour R. (2023). Systemic delivery of specific and efficient CRISPR/Cas9 system targeting HPV16 oncogenes using LL-37 antimicrobial peptide in C57BL/6 mice. J. Med. Virol. 95, e28934. doi: 10.1002/jmv.28934, PMID: 37403986

[B36] Khairuddin N. Blake S. J. Firdaus F. Steptoe R. J. Behlke M. A. Hertzog P. J.. (2014). *In vivo* comparison of local versus systemic delivery of immunostimulating siRNA in HPV-driven tumours. Immunol. Cell Biol. 92, 156–163. doi: 10.1038/icb.2013.75, PMID: 24217808

[B37] Kumar S. Jena L. Galande S. Daf S. Mohod K. Varma A. K. (2014). Elucidating molecular interactions of natural inhibitors with HPV-16 E6 oncoprotein through docking analysis. Genomics Inform. 12, 64–70. doi: 10.5808/GI.2014.12.2.64, PMID: 25031569 PMC4099350

[B38] Lao Y. H. Li M. Gao M. A. Shao D. Chi C. W. Huang D.. (2018). HPV oncogene manipulation using nonvirally delivered CRISPR/cas9 or natronobacterium gregoryi argonaute. Adv. Sci. (Weinh). 5, 1700540. doi: 10.1002/advs.201700540, PMID: 30027026 PMC6051382

[B39] Lau C. H. Suh Y. (2017). *In vivo* genome editing in animals using AAV-CRISPR system: applications to translational research of human disease. F1000Res 6, 2153. doi: 10.12688/f1000research.11243.1, PMID: 29333255 PMC5749125

[B40] Li X. Guo M. Hou B. Zheng B. Wang Z. Huang M.. (2021). CRISPR/Cas9 nanoeditor of double knockout large fragments of E6 and E7 oncogenes for reversing drugs resistance in cervical cancer. J. Nanobiotechnology. 19, 231. doi: 10.1186/s12951-021-00970-w, PMID: 34353334 PMC8340365

[B41] Li L. Hu S. Chen X. (2018). Non-viral delivery systems for CRISPR/Cas9-based genome editing: Challenges and opportunities. Biomaterials. 171, 207–218. doi: 10.1016/j.biomaterials.2018.04.031, PMID: 29704747 PMC5944364

[B42] Lin Y. Wagner E. Lachelt U. (2022). Non-viral delivery of the CRISPR/Cas system: DNA versus RNA versus RNP. Biomater Sci. 10, 1166–1192. doi: 10.1039/d1bm01658j, PMID: 35103261

[B43] Ling K. Dou Y. Yang N. Deng L. Wang Y. Li Y.. (2023). Genome editing mRNA nanotherapies inhibit cervical cancer progression and regulate the immunosuppressive microenvironment for adoptive T-cell therapy. J. Control Release. 360, 496–513. doi: 10.1016/j.jconrel.2023.07.007, PMID: 37423524

[B44] Ljubojevic S. Skerlev M. (2014). HPV-associated diseases. Clin. Dermatol. 32, 227–234. doi: 10.1016/j.clindermatol.2013.08.007, PMID: 24559558

[B45] Makarova K. S. Zhang F. Koonin E. V. (2017a). SnapShot: class 1 CRISPR-cas systems. Cell. 168, 946–946.e941. doi: 10.1016/j.cell.2017.02.018, PMID: 28235204

[B46] Makarova K. S. Zhang F. Koonin E. V. (2017b). SnapShot: class 2 CRISPR-cas systems. Cell. 168, 328–328.e321. doi: 10.1016/j.cell.2016.12.038, PMID: 28086097

[B47] Malecka K. A. Fera D. Schultz D. C. Hodawadekar S. Reichman M. Donover P. S.. (2014). Identification and characterization of small molecule human papillomavirus E6 inhibitors. ACS Chem. Biol. 9, 1603–1612. doi: 10.1021/cb500229d, PMID: 24854633 PMC4145632

[B48] Milla P. Dosio F. Cattel L. (2012). PEGylation of proteins and liposomes: a powerful and flexible strategy to improve the drug delivery. Curr. Drug Metab. 13, 105–119. doi: 10.2174/138920012798356934, PMID: 21892917

[B49] Miron-Barroso S. Domenech E. B. Trigueros S. (2021). Nanotechnology-based strategies to overcome current barriers in gene delivery. Int. J. Mol. Sci. 22, 8537. doi: 10.3390/ijms22168537, PMID: 34445243 PMC8395193

[B50] Mitchell M. J. Billingsley M. M. Haley R. M. Wechsler M. E. Peppas N. A. Langer R. (2021). Engineering precision nanoparticles for drug delivery. Nat. Rev. Drug Discov. 20, 101–124. doi: 10.1038/s41573-020-0090-8, PMID: 33277608 PMC7717100

[B51] Moody C. A. Laimins L. A. (2010). Human papillomavirus oncoproteins: pathways to transformation. Nat. Rev. Cancer. 10, 550–560. doi: 10.1038/nrc2886, PMID: 20592731

[B52] Munoz N. Bosch F. X. de Sanjose S. Herrero R. Castellsague X. Shah K. V.. (2003). Epidemiologic classification of human papillomavirus types associated with cervical cancer. N Engl. J. Med. 348, 518–527. doi: 10.1056/NEJMoa021641, PMID: 12571259

[B53] Naso M. F. Tomkowicz B. Perry W. L. Strohl W. R. (2017). Adeno-associated virus (AAV) as a vector for gene therapy. BioDrugs. Aug 31, 317–334. doi: 10.1007/s40259-017-0234-5, PMID: 28669112 PMC5548848

[B54] Nelson C. E. Gersbach C. A. (2016). Engineering delivery vehicles for genome editing. Annu. Rev. Chem. Biomol Eng. 7, 637–662. doi: 10.1146/annurev-chembioeng-080615-034711, PMID: 27146557

[B55] Noguchi H. Matsushita M. Kobayashi N. Levy M. F. Matsumoto S. (2010). Recent advances in protein transduction technology. Cell Transplant. 19, 649–654. doi: 10.3727/096368910X508744, PMID: 20525440

[B56] Noroozi Z. Shamsara M. Valipour E. Esfandyari S. Ehghaghi A. Monfaredan A.. (2022). Antiproliferative effects of AAV-delivered CRISPR/Cas9-based degradation of the HPV18-E6 gene in HeLa cells. Sci. Rep. 12, 2224. doi: 10.1038/s41598-022-06025-w, PMID: 35140292 PMC8828776

[B57] OhAinle M. Helms L. Vermeire J. Roesch F. Humes D. Basom R.. (2018). A virus-packageable CRISPR screen identifies host factors mediating interferon inhibition of HIV. Elife. Dec 6, 7. doi: 10.7554/eLife.39823, PMID: 30520725 PMC6286125

[B58] Ojesina A. I. Lichtenstein L. Freeman S. S. Pedamallu C. S. Imaz-Rosshandler I. Pugh T. J.. (2014). Landscape of genomic alterations in cervical carcinomas. Nature. 506, 371–375. doi: 10.1038/nature12881, PMID: 24390348 PMC4161954

[B59] Parfenov M. Pedamallu C. S. Gehlenborg N. Freeman S. S. Danilova L. Bristow C. A.. (2014). Characterization of HPV and host genome interactions in primary head and neck cancers. Proc. Natl. Acad. Sci. U.S.A. 111, 15544–15549. doi: 10.1073/pnas.1416074111, PMID: 25313082 PMC4217452

[B60] Park J. S. Kim E. J. Kwon H. J. Hwang E. S. Namkoong S. E. Um S. J. (2000). Inactivation of interferon regulatory factor-1 tumor suppressor protein by HPV E7 oncoprotein. Implication for the E7-mediated immune evasion mechanism in cervical carcinogenesis. J. Biol. Chem. 275, 6764–6769. doi: 10.1074/jbc.275.10.6764, PMID: 10702232

[B61] Perkins R. B. Guido R. S. Castle P. E. Chelmow D. Einstein M. H. Garcia F.. (2020). 2019 ASCCP risk-based management consensus guidelines for abnormal cervical cancer screening tests and cancer precursors. J. Low Genit Tract Dis. 24, 102–131. doi: 10.1097/LGT.0000000000000525, PMID: 32243307 PMC7147428

[B62] Ran F. A. (2017). Adaptation of CRISPR nucleases for eukaryotic applications. Anal. Biochem. 532, 90–94. doi: 10.1016/j.ab.2016.10.018, PMID: 27984015

[B63] Ran F. A. Cong L. Yan W. X. Scott D. A. Gootenberg J. S. Kriz A. J.. (2015). *In vivo* genome editing using Staphylococcus aureus Cas9. Nature. 520, 186–191. doi: 10.1038/nature14299, PMID: 25830891 PMC4393360

[B64] Rusan M. Li Y. Y. Hammerman P. S. (2015). Genomic landscape of human papillomavirus-associated cancers. Clin. Cancer Res. 21, 2009–2019. doi: 10.1158/1078-0432.CCR-14-1101, PMID: 25779941 PMC4417456

[B65] Sakuma T. Masaki K. Abe-Chayama H. Mochida K. Yamamoto T. Chayama K. (2016). Highly multiplexed CRISPR-Cas9-nuclease and Cas9-nickase vectors for inactivation of hepatitis B virus. Genes Cells. 21, 1253–1262. doi: 10.1111/gtc.12437, PMID: 27659023

[B66] Schiffman M. Doorbar J. Wentzensen N. de Sanjose S. Fakhry C. Monk B. J.. (2016). Carcinogenic human papillomavirus infection. Nat. Rev. Dis. Primers. 2, 16086. doi: 10.1038/nrdp.2016.86, PMID: 27905473

[B67] Schiller J. T. Castellsague X. Garland S. M. (2012). A review of clinical trials of human papillomavirus prophylactic vaccines. Vaccine 30 Suppl 5, F123–F138. doi: 10.1016/j.vaccine.2012.04.108, PMID: 23199956 PMC4636904

[B68] Small W. Jr. Bacon M. A. Bajaj A. Chuang L. T. Fisher B. J. Harkenrider M. M.. (2017). Cervical cancer: A global health crisis. Cancer 123, 2404–2412. doi: 10.1002/cncr.30667, PMID: 28464289

[B69] Sousa D. A. Gaspar R. Ferreira C. J. O. Baltazar F. Rodrigues L. R. Silva B. F. B. (2022). *In vitro* CRISPR/cas9 transfection and gene-editing mediated by multivalent cationic liposome-DNA complexes. Pharmaceutics. 14, 1087. doi: 10.3390/pharmaceutics14051087, PMID: 35631673 PMC9143451

[B70] Stanley M. Pinto L. A. Trimble C. (2012). Human papillomavirus vaccines–immune responses. Vaccine 30 Suppl 5, F83–F87. doi: 10.1016/j.vaccine.2012.04.106, PMID: 23199968

[B71] Strecker J. Jones S. Koopal B. Schmid-Burgk J. Zetsche B. Gao L.. (2019). Engineering of CRISPR-Cas12b for human genome editing. Nat. Commun. 10, 212. doi: 10.1038/s41467-018-08224-4, PMID: 30670702 PMC6342934

[B72] Symington L. S. Gautier J. (2011). Double-strand break end resection and repair pathway choice. Annu. Rev. Genet. 45, 247–271. doi: 10.1146/annurev-genet-110410-132435, PMID: 21910633

[B73] Tasset A. Bellamkonda A. Wang W. Pyatnitskiy I. Ward D. Peppas N.. (2022). Overcoming barriers in non-viral gene delivery for neurological applications. Nanoscale. 14, 3698–3719. doi: 10.1039/d1nr06939j, PMID: 35195645 PMC9036591

[B74] Van Hees M. Slott S. Hansen A. H. Kim H. S. Ji H. P. Astakhova K. (2022). New approaches to moderate CRISPR-Cas9 activity: Addressing issues of cellular uptake and endosomal escape. Mol. Ther. 30, 32–46. doi: 10.1016/j.ymthe.2021.06.003, PMID: 34091053 PMC8753288

[B75] Wang W. Arca E. Sinha A. Hartl K. Houwing N. Kothari S. (2022). Cervical cancer screening guidelines and screening practices in 11 countries: A systematic literature review. Prev. Med. Rep. 28, 101813. doi: 10.1016/j.pmedr.2022.101813, PMID: 35637896 PMC9142642

[B76] Wang J. Guo M. Wang Q. Dang J. Liu X. Jin Z. (2018). Blocking activity of the HPV18 virus in cervical cancer cells using the CRISPR/Cas9 system. Int. J. Clin. Exp. Pathol. 11, 4230–4235. doi: 10.3892/ol.2018.4230, PMID: 31949818 PMC6962800

[B77] Wang D. Zhang F. Gao G. (2020). CRISPR-based therapeutic genome editing: strategies and *in vivo* delivery by AAV vectors. Cell. 181, 136–150. doi: 10.1016/j.cell.2020.03.023, PMID: 32243786 PMC7236621

[B78] Wiley D. J. Douglas J. Beutner K. Cox T. Fife K. Moscicki A. B.. (2002). External genital warts: diagnosis, treatment, and prevention. Clin. Infect. Dis. 35, S210–S224. doi: 10.1086/342109, PMID: 12353208

[B79] Woodman C. B. Collins S. I. Young L. S. (2007). The natural history of cervical HPV infection: unresolved issues. Nat. Rev. Cancer. 7, 11–22. doi: 10.1038/nrc2050, PMID: 17186016

[B80] Xiong J. Tan S. Yu L. Shen H. Qu S. Zhang C.. (2021). E7-targeted nanotherapeutics for key HPV afflicted cervical lesions by employing CRISPR/Cas9 and poly (Beta-amino ester). Int. J. Nanomedicine 16, 7609–7622. doi: 10.2147/IJN.S335277, PMID: 34819726 PMC8606985

[B81] Xu M. Luo W. Elzi D. J. Grandori C. Galloway D. A. (2008). NFX1 interacts with mSin3A/histone deacetylase to repress hTERT transcription in keratinocytes. Mol. Cell Biol. Aug 28, 4819–4828. doi: 10.1128/MCB.01969-07, PMID: 18505829 PMC2493374

[B82] Yan J. Kang D. D. Dong Y. (2021). Harnessing lipid nanoparticles for efficient CRISPR delivery. Biomater Sci. Sep 14 9, 6001–6011. doi: 10.1039/d1bm00537e, PMID: 34115079 PMC8440433

[B83] Yin D. Ling S. Wang D. Dai Y. Jiang H. Zhou X.. (2021). Targeting herpes simplex virus with CRISPR-Cas9 cures herpetic stromal keratitis in mice. Nat. Biotechnol. May 39, 567–577. doi: 10.1038/s41587-020-00781-8, PMID: 33432198 PMC7611178

[B84] Yoshiba T. Saga Y. Urabe M. Uchibori R. Matsubara S. Fujiwara H.. (2019). CRISPR/Cas9-mediated cervical cancer treatment targeting human papillomavirus E6. Oncol. Lett. 17, 2197–2206. doi: 10.3892/ol.2018.9815, PMID: 30675284 PMC6341785

[B85] Zetsche B. Gootenberg J. S. Abudayyeh O. O. Slaymaker I. M. Makarova K. S. Essletzbichler P.. (2015). Cpf1 is a single RNA-guided endonuclease of a class 2 CRISPR-Cas system. Cell. 163, 759–771. doi: 10.1016/j.cell.2015.09.038, PMID: 26422227 PMC4638220

[B86] Zhang Z. Baxter A. E. Ren D. Qin K. Chen Z. Collins S. M.. (2024). Efficient engineering of human and mouse primary cells using peptide-assisted genome editing. Nat. Biotechnol. Feb 42, 305–315. doi: 10.1038/s41587-023-01756-1, PMID: 37095348 PMC11230135

[B87] Zhang I. Hsiao Z. Liu F. (2021). Development of genome editing approaches against herpes simplex virus infections. Viruses. 22, 13. doi: 10.3390/v13020338, PMID: 33671590 PMC7926879

[B88] Zhen S. Hua L. Liu Y. H. Gao L. C. Fu J. Wan D. Y.. (2015). Harnessing the clustered regularly interspaced short palindromic repeat (CRISPR)/CRISPR-associated Cas9 system to disrupt the hepatitis B virus. Gene Ther. 22, 404–412. doi: 10.1038/gt.2015.2, PMID: 25652100

[B89] Zhen S. Hua L. Takahashi Y. Narita S. Liu Y. H. Li Y. (2014). *In vitro* and *in vivo* growth suppression of human papillomavirus 16-positive cervical cancer cells by CRISPR/Cas9. Biochem. Biophys. Res. Commun. 450, 1422–1426. doi: 10.1016/j.bbrc.2014.07.014, PMID: 25044113

[B90] Zhen S. Liu Y. Lu J. Tuo X. Yang X. Chen H.. (2020a). Human papillomavirus oncogene manipulation using clustered regularly interspersed short palindromic repeats/Cas9 delivered by pH-sensitive cationic liposomes. Hum. Gene Ther. 31, 309–324. doi: 10.1089/hum.2019.312, PMID: 31973584

[B91] Zhen S. Lu J. Liu Y. H. Chen W. Li X. (2020b). Synergistic antitumor effect on cervical cancer by rational combination of PD1 blockade and CRISPR-Cas9-mediated HPV knockout. Cancer Gene Ther. 27, 168–178. doi: 10.1038/s41417-019-0131-9, PMID: 31455836

[B92] Zhen S. Lu J. J. Wang L. J. Sun X. M. Zhang J. Q. Li X.. (2016). *In vitro* and *in vivo* synergistic therapeutic effect of cisplatin with human papillomavirus16 E6/E7 CRISPR/cas9 on cervical cancer cell line. Transl. Oncol. 9, 498–504. doi: 10.1016/j.tranon.2016.10.002, PMID: 27816686 PMC5094426

[B93] Zhen S. Qiang R. Lu J. Tuo X. Yang X. Li X. (2023). CRISPR/Cas9-HPV-liposome enhances antitumor immunity and treatment of HPV infection-associated cervical cancer. J. Med. Virol. 95, e28144. doi: 10.1002/jmv.28144, PMID: 36121194

